# Interactive Multimodal Ambulatory Monitoring to Investigate the Association between Physical Activity and Affect

**DOI:** 10.3389/fpsyg.2012.00596

**Published:** 2013-01-18

**Authors:** U. W. Ebner-Priemer, S. Koudela, G. Mutz, M. Kanning

**Affiliations:** ^1^Karlsruhe Institute of TechnologyKarlsruhe, Germany; ^2^Central Institute of Mental HealthMannheim, Germany; ^3^University of CologneCologne, Germany; ^4^University of StuttgartStuttgart, Germany

**Keywords:** ambulatory monitoring, e-diary, interactive assessment, physical activity

## Abstract

Although there is a wealth of evidence that physical activity has positive effects on psychological health, a large proportion of people are inactive. Data regarding counts, steps, and movement patterns are limited in their ability to explain why people remain inactive. We propose that multimodal ambulatory monitoring, which combines the assessment of physical activity with the assessment of psychological variables, helps to elucidate real world physical activity. Whereas physical activity can be monitored continuously, psychological variables can only be assessed at discrete intervals, such as every hour. Moreover, the assessment of psychological variables must be linked to the activity of interest. For example, if an *inactive and overweight person* is physically active once a week, psychological variables should be assessed during this episode. Linking the assessment of psychological variables to episodes of an activity of interest can be achieved with interactive monitoring. The primary aim of our interactive multimodal ambulatory monitoring approach was to intentionally increase the number of e-diary assessments during “active” episodes. We developed and tested an interactive monitoring algorithm that continuously monitors physical activity in everyday life. When predefined thresholds are surpassed, the algorithm triggers a signal for participants to answer questions in their electronic diary. Using data from 70 participants wearing an accelerative device for 24 h each, we found that our algorithm quadrupled the frequency of e-diary assessments during the activity episodes of interest compared to random sampling. Multimodal interactive ambulatory monitoring appears to be a promising approach to enhancing our understanding of real world physical activity and movement.

## Introduction

Despite the positive effects of physical activity on health that have been demonstrated in the scientific literature (Healy et al., 2008; Physical Activity Guidelines Committee, 2008; Mead et al., 2009; Shiroma and Lee, 2010; Poole et al., 2011; Wichers et al., 2012) and despite that these positive effects are known to the community, a large number of people remain inactive compared to current guidelines (Haskell et al., 2007; Troiano et al., 2008). Unfortunately, data regarding counts, steps, and movement patterns have a limited ability to explain why people are inactive. To explain “inactive behavior,” additional parameters must be taken into account such as motivational processes, habits, and/or affective states. Interestingly, a positive association between physical activity and affective states has been claimed for a long time (Schlicht, 1994; Arent et al., 2000; Netz et al., 2005). However, the methodological quality of these studies is mixed.

In the following, we will report on methodological considerations how to assess the relation between physical activity and affective states, emphasizing (a) objective assessment of physical activity, (b) assessment in real life, and (c) problems of different time courses when using multimodal assessment. All these considerations sum up in our recommendation to use interactive multimodal ambulatory assessment. After this introduction, we will report (a) how we developed the interactive assessment approach and (b) how it was validated. Specifically, we will report how we tested our primary aim of the interactive multimodal ambulatory monitoring approach, namely to intentionally increase the number of e-diary assessments during rare “active” episodes. We will conclude the paper with limitations and future prospects.

### Self-reports vs. objective assessments

Multiple studies, investigating the effects of physical activity on physiological and psychological health, have been criticized for using self-reports to measure physical activity. For example, Shiroma and Lee (2010) summarizing 34 prospective studies with approximately 500,000 subjects in a review article state in their limitation section that most available data comes from observational studies with self-reported physical activity. This method might be limited in precision, and they further highlight that motion sensors (e.g., accelerometers) allow for physical activity and sedentary behavior in free-living populations to be assessed with greater accuracy and precision. Similar arguments have been made by Haskell et al. (2007), Schwerdtfeger et al. (2008), Poole et al. (2011), Wichers et al. (2012).

This methodological concern has been confirmed by two systematic reviews (Prince et al., 2008; Adamo et al., 2009) showing substantial discrepancies and only moderate correlations between objective assessments of physical activity and subjective reports of physical activity. Prince et al. (2008) compared subjectively (self-reported; e.g., questionnaire, diary) and objectively (directly measured; mostly accelerometry) assessed physical activity in adults. An analysis of 187 studies revealed only low-to-moderate correlations between self-reported and direct measures, with a mean of 0.37 (SD = 0.25; range −0.71 to 0.98). Similarly, in a systematic review on physical activity in children (83 studies), Adamo et al. (2009) found low-to-moderate associations (−0.56 to 0.89) between indirect and direct measures. Consequently, Adamo et al. (2009) and Prince et al. (2008) question the widespread approach of justifying the use of the more cost-effective self-report methods through correlations between indirect and direct measures.

In addition, there is considerable variability in study findings regarding the fulfillment of physical activity guidelines, especially when using different assessment methods (Reilly et al., 2008). Whereas national surveillance of pediatric physical activity in the UK showed that public health exercise-related targets were being exceeded by over 75% (e.g., in the Scottish Health Survey 2003) using subjective (parental) reports of physical activity, comparable UK studies using accelerometry as a measure of physical activity revealed that less than 5% of children and adolescents were meeting the target of 60 min of moderate-to-vigorous physical activity per day (Reilly et al., 2008). Using objective data obtained with accelerometers from a representative sample of the U.S. population (6329 participants), Troiano et al. (2008) reported that only 8% of adolescents achieved the recommended 60 min/day of physical activity, whereas among adults, adherence to the recommendation to obtain 30 min/day of physical activity was less than 5%.

Neither systematic review (Prince et al., 2008; Adamo et al., 2009) nor studies on the fulfillment of physical activity guidelines, support the “quick-and-dirty” retrospective questionnaire approach; instead, they indicate that actual physical activity is not accurately assessed by subjective self-reports. However, it has to be mentioned that retrospective questionnaire approaches in epidemiology show consistently negative relations between amount of physical activity and cardiovascular diseases or diabetes. Clearly, retrospective questionnaire approaches might be less accurate, but they still show some kind of validity.

### Monitoring in everyday life

In addition to using objective and multimodal monitoring methods, the investigation of physical activity in everyday life has clear benefits due to the distinction between performance and capacity: *“Performance is about what people do in their daily life … and capacity is about what people can do in an optimal environment …”* (Bussmann and Ebner-Priemer, 2011). As the literature has shown, the relationship between capacity and performance parameters is generally weak or absent. Therefore, behavior cannot be predicted from capacity tests, and direct measurements of performance are necessary to obtain insight into what people do in their daily life (Bussmann and Ebner-Priemer, 2011). However, there is also a downside to studying everyday life. Compared to a laboratory, the setting and behavior in everyday life cannot be controlled. Isolation of stimuli, manipulation of independent variables, rigorous control of extraneous variables and control of measurement errors are clearly difficult if not impossible (Fahrenberg, 1996). This is especially problematic when the phenomenon of interest is rare in everyday life. Taking into account that many people are too inactive in everyday life, the ability to investigate relationships between physical activity and psychological variables such as affective state is limited. From a statistical point of view, a minimal number (or variance) of active episodes is required to obtain a correlation.

### Multimodal monitoring: The problem of different time courses when combining the assessment of physical activity and affective states

More recent studies have used activity monitors and electronic diaries in assessing the relation between physical activity and mood (Schwerdtfeger et al., 2008; Kanning et al., 2012; Wichers et al., 2012). For example, Schwerdtfeger et al. (2008) used accelerometers and electronic diaries to investigate the association between physical activity and affective states prospectively. Mixed model analyses revealed a significant association between energetic arousal/positive affect and preceding physical activity. However, no relation between physical activity and negative affect was found. Similar findings have been reported by Wichers et al. (2012), who investigated the time-lagged association between daily life physical activity and affect using paper-pencil diaries. Their analysis revealed a significant increase in positive affect following a reported increase in physical activity. However, they did not find an effect of physical activity on negative affect. Kanning et al. (2012) investigated the relationship between actual physical activity and affective states in 44 university students using 24-h accelerometry and electronic diaries. Multilevel analyses showed that physical activity prospectively predicted valence and calmness. The more the subjects were physically active during the preceding 10 min, the more they felt well and satisfied (valence domain), but they also felt agitated and tense (calmness domain). This unexpected discrepancy, between people reporting being more agitated and tense and the findings reported above that physical activity had no effect on negative affect (Schwerdtfeger et al., 2008; Wichers et al., 2012), might be explained by different time courses of the variables of interest.

This idea can be explained with a very simple example. Imagine a very overweight person who had been inactive for a long period over time. After advice from his/her physician, this person starts performing some kind of sport, e.g., running. He might feel uncomfortable, ridiculous, and ashamed while running the first time after a long period. However, he also might feel proud, relieved, and positively exhausted half an hour after having performed running. Similar examples can be found in a variety of situations, e.g., running during bad weather, running when someone is tired, and getting up very early to do sports. The underlying idea is that the time courses of physical activity, positive and negative affective states might not be parallel but may be shifted. Therefore, we propose that multimodal monitoring, in which physical activity and movement are assessed in conjunction with psychological variables and the specific context of the activity and movement, might be necessary to understand real world physical activity and movement. An initial validation of the idea of shifted time courses has been reported by Schwerdtfeger et al. (2010). They took a closer look at the temporal relation between affective states and physical activity. They reanalyzed 12-h ambulatory monitoring data from 124 healthy volunteers from a previously published dataset (Schwerdtfeger et al., 2008). Multilevel analyses revealed that negative affective states were not related to an increase in physical activity in the following 1 or 5 min. However, later on, significant effects of negative affective states on physical activity were detected (15 and 30 min after the affective state assessment).

## Developing the Interactive Assessment Approach

### Interactive multimodal ambulatory monitoring: Methodological considerations

In summary, to understand the relationships between actual physical activity and affective states, a multimodal monitoring of everyday life using objective and real-time methods seems to be advantageous. Unfortunately, assessing psychological variables and context in everyday life can be difficult. Whereas physical activity can be monitored continuously with a high sampling frequency, psychological variables can only be assessed in discrete intervals such as every 30 min or every hour. Even more daunting, the assessment of psychological variables must be linked temporally to the physical phenomena of interest and not be purely random. Considering our example of the “inactive and overweight person” who might be physically active twice a month, the psychological variables should be assessed during or immediately after this active episode to obtain some variance in physical activity and thereby enable some kind of correlation.

**Figure 1:**
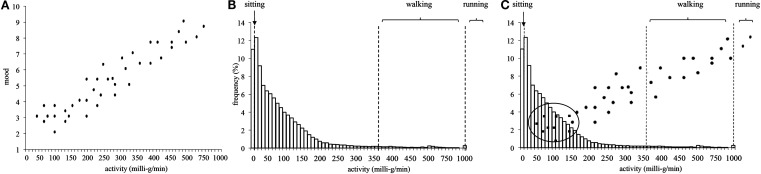
**(A)** Hypothetical data showing a positive relation between physical activity and affective state; **(B)** frequencies of physical activity (70 students, 24 h) aggregated over 10-min episodes. Please note: *X*-axis intervals increase from 10 to 50 mg intervals after 500 mg/min for graphical reasons; **(C)** combination of the hypothetical and empirical data.

This issue is explained in greater detail in Figure 1. Figure 1A depicts a hypothetical relationship between physical activity and affective state. It is assumed to be a positive correlation, showing increasing affective state values with increasing physical activity. Figure 1B shows empirical data. Physical activity was assessed in 70 undergraduate students from a German university (University of Stuttgart; the 70 students are a subsample of the data reported in Kanning et al., 2012; informed consent and institutional ethical approval were obtained). Physical activity was measured continuously for 24 h using a three-way accelerometer (varioport-E; Becker Meditech, Germany), which was attached at the participant’s hip. Acceleration, measured in milli-g (mg), was separated offline into AC and DC components by an Finite Impulse Response (FIR) digital filter with a cut-off frequency at 0.5 Hz. The raw signal, DC-values, and rectified AC-values were averaged across data points for each minute for the 24 h. All offline analyses and artifact checks were performed by the interactive software package “Freiburg Monitoring System” according to a published procedure (Myrtek, 2004). Rectified AC-values of the three channels were aggregated for 10-min episodes. The frequencies of these 10-min episodes are shown in Figure 1B (with sleeping time being excluded beforehand). The *x*-axis unit is mg. For comparison, jogging episodes entail about 1000 mg/min, walking episodes about 350 mg/min, and pure sitting episodes about 10 mg/min. Note that the *x*-axis intervals increase from 10 to 50 mg intervals after 500 mg/min for graphical reasons. What is evident from Figure 1B is that episodes of high physical activity are rare in this sample. This finding is not surprising in light of the complaints regarding low levels of physical activity in the general population (Haskell et al., 2007; Reilly et al., 2008; Troiano et al., 2008). The underlying problem becomes apparent when combining the hypothetical relation (Figure 1A) with the empirical data (Figure 1B) as in Figure 1C. When assessing affective states purely randomly over the day, the chance of obtaining affective states data during an episode of high physical activity is decidedly small. For example, in Figure 1B, the chance of having an episode above 350 mg (≈ 10 min of pure walking during a 10-min episode) is less than 3%. A relation between physical activity and affective state (or a reduction of negative affective state due to physical activity) might well remain undetected in the statistical analysis purely due to the low number of episodes with high physical activity.

**Figure 2:**
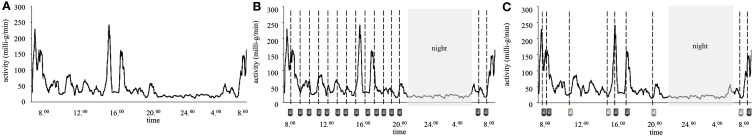
**(A)** Time course over 24 h of physical activity (10 min moving average) in a single subject; **(B)** combined with a fixed e-diary assessment, or **(C)** combined with an interactive e-diary assessment.

Solving this problem is possible by changing the affective state assessment strategy from a random or fixed (e.g., hourly) mode to an interactive assessment mode. This approach is depicted in Figure 2. Figure 2A shows a time course over 24 h of physical activity (moving average over 10 min) in one subject. Over the day, there are several peaks of physical activity. During the night, the physical activity is low. An hourly assessment of affective states, as illustrated in Figure 2B, would coincide with some peaks of physical activity but would miss others. From a statistical point of view, maximization of the variance is favored. In our example, this would mean obtaining e-diary assessments of the affective state for several episodes of high physical activity as well as for episodes of low physical activity (see Figure 2C). This can be achieved with an interactive monitoring approach. In interactive monitoring, the phenomenon of interest (in our case physical activity, or more technically acceleration) is monitored continuously and analyzed continuously in real-time during everyday life. Interesting episodes are detected in real-time and are used as triggers for the assessment of psychological variables and contextual information. This results in the intentional oversampling of e-diary assessments that are related to episodes with high physical activity compared to the natural occurrence of these episodes. We developed an algorithm that continuously monitors physical activity in everyday life and triggers e-diary assessments when predefined thresholds are surpassed.

### The interactive algorithm

The idea of interactive monitoring is not entirely new, as Myrtek (2004) has developed and worked with this methodology. He used interactive monitoring with real-time analysis of physical activity and physiological signals to detect emotional and physical influences on physiological processes, so called additional heart rate. In short, he monitored heart rate and physical activity in daily life and separated out in real-time heart rate increases caused by physical activity. Any remaining additional heart rate increase was assumed to indicate momentary emotional activation or mental load. The recorder/analyzer was programmed to trigger a hand-held PC, which in turn signals the participant to self-report on momentary activity, the situation, and their emotions. This occurs when the additional heart rate exceeds a certain threshold. Control periods were obtained by using randomly interspersed trigger signals. The algorithm was used and validated in a series of studies based on many different samples and around 1,300 participants (Myrtek, 2004). Unfortunately, applications of interactive monitoring in everyday life are, aside from Myrtek, still extremely rare. As we used the methodology of Myrtek in an earlier study (Ebner, 2004; Ebner-Priemer et al., 2007) and intensively tested Myrtek’s algorithm (Ebner, 2004), the interactive algorithm proposed in this paper is strongly influenced by Myrtek’s work.

#### Hardware

To our knowledge, commercial devices that offer the additional possibility of online analysis, and are therefore known as recorder-analyzer systems, are extremely rare. Ebner-Priemer and Kubiak (2007) list three different systems that support interactive monitoring: the varioport-B, the varioport-E (both from Becker Meditec, Karlsruhe, Germany), and the g.MOBIlab (g.tec, Graz, Austria). MATLAB, C, and Simulink can be used for g.MOBIlab, and the programming language SPIL can be used for the vitaport/varioport family. We selected the varioport-E because of his minimal size and its good cost-benefit ratio. The varioport is a modular, multi-purpose device that can be flexibly adapted to a range of research questions. The varioport-E has an integrated three-dimensional accelerative sensor (ADXL330) and is capable of measuring one additional physiological signal (such as ECG). Because up to now the varioport-E has not been used to investigate the relationship between physical activity and affective states, we initially screened the validity of the varioport-E. We tested three different accelerative devices on a commercial treadmill (Life Fitness 95T Inspire). Two varioport-Es were fixed on the hip (left and right), one actiheart^®^ monitor (Firmware H90.65; Camntech, Cambridge, UK) was attached to the chest, and one Actigraph (GT1M Firmware 7.5.0; The Actigraph, Pensacola, FL, USA) was fixed on the left hip with a belt. Six subjects ran at five different speeds (0, 4.6, 9.2, 13.8, 19.4 km/h) for at least 60 s (only 30 s for the 19.4 km/h). For the actigraph, the two channels were aggregated. For the varioport, all three channels were aggregated.

**Figure 3:**
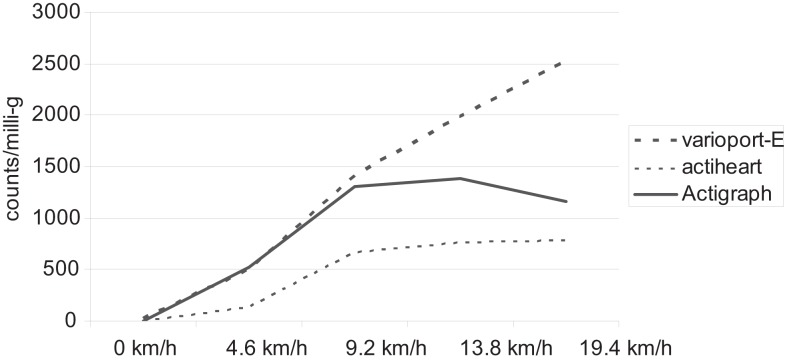
**Ceiling effects of actiheart^®^ and actigraph in comparison to varioport-E by increasing running speeds**.

Whereas both varioport-Es indicated increasing values for the increasing speeds, both of the other devices showed ceiling effects at the higher speeds as shown in Figure 3. [varioport-Es: 0 km/h = 22/23 mg; 4.6 km/h = 490/503 mg; 9.2 km/h = 1415/1459 mg; 13.8 km/h = 1893/1984 mg; 19.4 km/h = 2382/2523 mg; actiheart^®^: 0 km/h = 0 counts; 4.6 km/h = 133 counts; 9.2 km/h = 663 counts; 13.8 km/h = 761 counts; 19.4 km/h = 781 counts; Actigraph GT1M: 0 km/h = 2 counts; 4.6 km/h = 526 counts; 9.2 km/h = 1307 counts; 13.8 km/h = 1386 counts; 19.4 km/h = 1158 counts). Accordingly the (pooled) within-subject correlation between speed and physical activity (mg; counts) was descriptively higher for both varioport-Es (*r* = 0.99; *r* = 0.98) compared to the actiheart^®^ (*r* = 0.91) and the Actigraph GT1M (*r* = 0.81).

#### Adaptive thresholds

Two basic components were adopted from the algorithm developed by Myrtek. (1) Three events were defined as triggers for the electronic diary: (I) an activity threshold, (II) an inactivity threshold, and (III) a time-limit threshold. (2) The thresholds were defined to be adaptive: in cases of low physical activity across the whole assessment period (≈ very inactive subject), both the activity threshold and the inactivity threshold decrease from one e-diary assessment to the next. This was done to obtain e-diary assessments during the most active and the least active episodes in each individual independently of the absolute amount of activity. The same procedure was used for very active people. If too many episodes of activity are detected, the thresholds for activity episodes and inactivity episodes increase to obtain e-diary assessments during the most active and the least active episodes in each individual.

#### Basic decisions

Before programming the algorithm for detecting the interesting episodes of physical activity in everyday life, a few decisions had to be made. First, thresholds for both activity episodes and non-activity episodes had to be set. Such a decision can be made (a) theoretically, e.g., based on findings regarding what amount of physical activity leads to acute changes in physiology or affective states; (b) empirically, e.g., according to the usual frequency of activity episodes in everyday life; or (c) from a time-based-design perspective. In electronic diary research, the time-based design is a crucial part of the research design. The time-based design is defined by the number of assessment points, the intervals between assessment points, and the total length of the assessment period (Ebner-Priemer and Sawitzki, 2007). The thresholds were defined using a combination of theoretical, empirical, and time-based-design considerations. First, we assumed that relatively short time frames would be most suitable for analyzing the effects of actual activities of daily life, which are mostly performed in short time periods. Schwerdtfeger et al. (2008) compared the correlation between physical activity and affective states using three different time frames (1, 5, 15 min). The correlation between 15-min averages of physical activity and affective states was higher compared to those of the 1- or 5-min averages. With the idea of integrating actual physical activities of everyday life instead of only athletic activities, this finding, in combination with the guideline that being physically active for a minimum of 10 min leads to health benefits (Haskell et al., 2007), led us to work with episodes of physical activity 10 min in duration. To obtain a minimum number of e-diary assessments per day, we set the number of assessments to 10. We excluded diary assessments during the night (sleeping time: 21.00–8.00). Given a minimum of 10 assessments per 13 daytime hours, we limited the time interval between e-diary assessments to a minimum of 40 min and a maximum of 100 min. This means that after an e-diary assessment, there will be no further e-diary assessment for the next 40 min regardless of any activity. If the algorithm is not able to detect an activity or inactivity episode between 40 and 99 min after the last e-diary assessment, the next e-diary assessment will be triggered at minute 100 (=time-limit threshold). Furthermore, we set the rate for activity/inactivity episodes to 1:1.

#### Deriving the thresholds from empirical data

We reanalyzed a subsample of 50 healthy subjects from a previously published study (Ebner-Priemer et al., 2007; informed consent and ethical approval were obtained). In this dataset, all participants underwent 24-h ambulatory monitoring of physical activity and ECG. Additionally, an individual reference pattern standard protocol with a fixed sequence of postures and movements (sitting upright, sitting while leaning forward, sitting while leaning backward, standing, lying back, lying on the right side, lying on the left side, walking) was recorded. Subsequently, multivariate within-subjects analyses and pattern similarity coefficients were used for the detection and labeling of an actual segment; that is, a hierarchical strategy was applied that classifies postures and subsequently uses reference patterns to discriminate between subsets of activities (Fahrenberg et al., 1997). Subjects carried a portable Vitaport II physiological digital recorder (Becker Engineering, Karlsruhe, Germany) and a palmtop computer (Psion 3 a, London, UK) for 24 h in their everyday lives. Physical activity was sampled at 32 Hz by two accelerative three-dimensional sensors placed on the chest below the clavicle and on the thigh above the knee. Rectified AC-values from the three acceleration channels at the chest were aggregated for 1-min episodes.

To derive a threshold for activity and inactivity episodes, we used the above-mentioned data set, excluded the night, and searched for the intensity of episodes 10 min in duration (moving average over 1-min segments) with a frequency of at least five per 24 h. This resulted in a cut-off value of 220 mg/min. Given the finding that walking is (on average) about 350 mg, a cut-off threshold of 220 mg would mean that subjects during such an episode walked at least 6 min during the past 10 min, which seems feasible for an everyday life approach. As we obtained a high number of inactivity episodes during everyday life in our dataset, we set the inactivity threshold to 10 mg/min, which is equivalent to the acceleration of sitting (on average). In sum, the constituent parts of the algorithm are as follows.

– (adaptive) threshold for activity episode: >220 mg (10 min moving average)
– (adaptive) threshold for inactivity episode: <10 mg (10 min moving average)
– aimed rate for activity/inactivity episodes: 1:1
– minimum and maximum time interval between e-diary assessments: 40–100 min
– sleeping time: 21.00–8.0

#### The program

The algorithm was programmed in SPIL for varioport and basically does the following:

(1) Calculates global activity (raw data for each axes, sampled at 32 Hz, were converted into mg, high pass filtered (>0.1 Hz), rectified, and smoothed by a moving average of 1 Hz. The three channels were aggregated using vector addition).
(2) Aggregates the signal over time from 32 Hz to 10-min segments (moving averages).
(3) Sends triggers for the e-diary when (a) the threshold for activity is surpassed, it is not sleeping time, and the last trigger was sent during the last 40 to 100 min, when (b) the threshold for inactivity was reached, it is not sleeping time, and the last trigger was sent during the last 40–100 min, or when (c) the last trigger was sent 100 min ago.
(4) The frequency of triggers is stored, and the ratio between inactivity and activity episode-based triggers is calculated. If there have been at least six triggers and the activity/inactivity trigger ratio is above 2.05, then the thresholds for activity and inactivity episodes are increased by 5% (which is decreasing the likelihood of upcoming activity episodes and increasing the likelihood of upcoming inactivity episodes). If there have been at least six triggers and the inactivity/inactivity trigger ratio is below 0.95, then the thresholds for activity and inactivity episodes are decreased by 5% (which is increasing the likelihood of upcoming activity episodes and decreasing the likelihood of upcoming inactivity episodes). If there have been at least six triggers and the current trigger is a time-limit trigger, then the threshold for activity episodes is decreased by 5% and the threshold for inactivity episodes is increased by 5% (which is increasing the likelihood of upcoming activity episodes and increasing the likelihood of upcoming inactivity episodes).

## A Validation Study of the Interactive Algorithm

We tested the algorithm offline in a dataset from 70 participants (which was described above; the 70 students were a subsample of the data reported in Kanning et al., 2012). Physical activity was measured continuously for 24 h using a three-way accelerometer (varioport-E; Becker Meditech, Germany), which was attached at the participant’s hip. To analyze the effectiveness of the algorithm, we compared the frequency of episodes (10 min) above the selected activity threshold (>220 mg/min), between the selected activity and inactivity threshold (>10 and <220 mg/min), and below the selected inactivity threshold (<10 mg/min). A purely random sampling strategy for the e-diary assessment would trigger e-diary assessments in only 9.3% of episodes above the selected activity threshold of >220 mg/min and in 9.4% of episodes below the selected inactivity threshold of <10 mg/min. Thus, most triggers (81.3%) would be sent during mixed episodes (>10 and <220 mg/min; see also Table 1).

**Table 1:** **Percentage of episodes revealed by an random or interactive sampling separated into three categories: above the selected activity threshold, between the selected activity and inactivity threshold, and below the selected inactivity threshold (please note, that the upper line “random sampling” corresponds to the white bars in Figure 4 and the lower line ”interactive sampling” to the gray bars)**.

	<10 mg/min (%)	>10 and <220 mg/min (%)	>220 mg/min (%)
Random sampling	9.4	81.3	9.3
Interactive sampling	30	32	38

**Figure 4:**
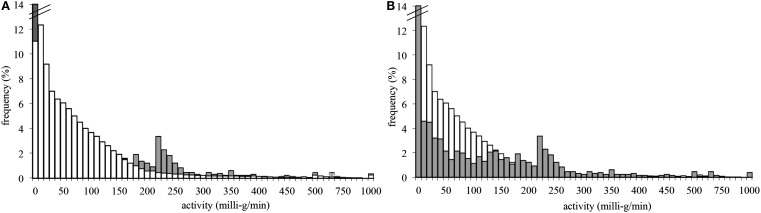
**Frequency of physical activity (70 students, 24 h) aggregated over 10-min episodes as revealed by random (white bars) or interactive (gray bars) e-diary assessment**. Please note that both graphs show the same data. In the first graph **(A)**, the interactive sampling is in the back, whereas in the second graph **(B)**, the random sampling is in the back. Furthermore, *X*-axis intervals increase from 10 to 50 mg intervals after 500 mg/min for graphical reasons.

Using the proposed interactive sampling strategy, the frequency of e-diary assessments during episodes above the activity threshold of >220 mg/min quadrupled compared to a random e-diary sampling, resulting in a frequency of 38% (see also Table 1). Similar results were found for episodes below the selected inactivity threshold, the frequency of which increased to 30%. Accordingly, the frequency of mixed episodes (>10 and <220 mg/min) decreased from 81.3 to 32%. The results are shown in Figure 4 in greater detail. The white bars represent a random e-diary assessment, whereas the gray bars represent interactive sampling. Note that both graphs in Figure 4 show the same data. In the first graph, the random sampling is in the front, whereas in the second graph, the interactive sampling is in the front. Furthermore, note that the first bar of the interactive sampling was cut from 30 to 14% for graphical reasons. Figure 4 shows the altered distribution of activity episodes during e-diary assessments. The frequency of inactivity episodes (according to our threshold) increased tremendously, the frequency of medium or mixed episodes between 10 and 220 mg/min decreased, whereas the frequency of episodes above our threshold of 220 mg/min increased. As the thresholds were adaptive, a low number of activity episodes in a subject led to a lowering of the activity threshold in this subject. Accordingly, the frequency of episodes just below 220 mg/min, especially between 180 and 210 mg/min, increased, as shown in Figure 4.

## Conclusion; Limitations and Future Prospects

The primary aim of our interactive multimodal ambulatory monitoring approach was to intentionally increase the number of e-diary assessments during “active” episodes. This goal was achieved, as we quadrupled the frequency of e-diary assessments above the selected activity threshold compared to a random sampling approach. In other words, the proposed interactive algorithm provided an advantageous distribution that might be much better suited, compared to a random sampling, to revealing relationships between activity and affective states in everyday life. We propose that multimodal interactive ambulatory monitoring of everyday life behaviors seems to be a promising approach to enhancing our understanding of real world physical activity and movement.

Several limitations should be mentioned. First, the ability to improve the distribution is limited by the actual frequency of active episodes. For example, when assessing a bedfast patient, no algorithm will be able to find an actual active episode. Although our algorithm uses adaptive thresholds, which are not fixed to absolute values but look for the most active episodes in each subject, the algorithm will work only in people with at least some degree of activity. With “extremely sedentary” and bedfast patients, the algorithm does not provide any advantages.

We used a pragmatic approach to develop the proposed algorithm, making some basic assumptions and borrowing operational procedures from the additional heart rate algorithm developed by Myrtek (2004). We cannot rule out that other assumptions might work better for investigating relationships between physical activity and affective states. Favorable results might be achieved by changing (increasing or decreasing) the 10-min moving average time interval, the activity:inactivity rate of 1:1, or the activity and inactivity thresholds. Although our algorithm improved the distribution by quadrupling the number of episodes above our set activity threshold, further work is necessary. Another questions remains regarding the time sampling strategy. We assessed affect directly after a 10 min episode, if a certain threshold was exceeded. Another promising approach might be, to assess affect 5 min after terminating the physical activity episodes. This would answer questions regarding how participants feel after during physical activity and not during. Clearly, more studies are needed.

The most convincing evidence for the effectiveness of an interactive monitoring might seem to show “higher” correlations between physical activity and affect when using interactive monitoring compared to randomly distributed or fixed-interval assessments (such as every hour) in a single dataset. Unfortunately, showing both correlation on a single data set is not possible. The e-diary assessment has to follow one specific rule (interactive, random, or fixed), and results are based on this specific assessment rule. Therefore, it’s not possible recording and analyzing a single dataset using different sampling strategies. Because of that, we focused on improving the distribution in our analysis, to show potential benefits of interactive assessments Future studies might randomize participants into two groups, one with interactive assessment and one with random/fixed assessment. This might enable to compare correlations between physical activity and affect using different sampling strategies.

The proposed interactive multimodal ambulatory monitoring procedure is not limited to investigating the relationship between physical activity and affective states. A similar argument can be made for movement abnormalities in everyday life. Balance problems detectible by movement monitoring can be caused by a certain physical condition or by uneven pavement. Real-time analysis of balance problems may trigger e-diaries. Assessing the context (for example, with a simple photograph of the pavement) can help to decide if a certain episode of movement abnormality should be attributed to a physical condition. Considerations regarding a real-time movement pattern classification system are also possible. If the software is unable to fit a special movement (e.g., bowling) to a fixed standard set of movement patterns because it is not yet part of the classification system, interactive monitoring with real-time classification might detect the unclassifiable pattern in real-time and ask the subject about the nature of his current activity. In a further step, the interactive monitoring system might even integrate this movement pattern into the individual classification system, allowing the completeness and accuracy of the movement classification to be continually improved.

## Conflict of Interest Statement

The authors declare that the research was conducted in the absence of any commercial or financial relationships that could be construed as a potential conflict of interest.
